# Astilbin Alleviates Gouty Arthritis via Regulating NLRP3 Inflammasome and NF-κB Signaling Pathway: A Comprehensive Study on In Vitro and In Vivo Experimental Models

**DOI:** 10.3390/nu18142360

**Published:** 2026-07-18

**Authors:** Xiaoxi Zhang, Gaoyang Fu, Xinyu Zhao, Yan Huang, Fenfen Li, Daozong Xia

**Affiliations:** 1 Department of Food Science and Nutrition, School of Pharmaceutical Sciences, Zhejiang Chinese Medical University, Hangzhou 310053, China; 20201059@zcmu.edu.cn (X.Z.); 15715431779@163.com (G.F.); zhaoxinyu10211021@163.com (X.Z.); huangyanoct@163.com (Y.H.); ncusklifenfen@163.com (F.L.); 2 Academy of Chinese Medical Sciences, Zhejiang Chinese Medical University, Hangzhou 310053, China

**Keywords:** gouty arthritis, astilbin, NLRP3, NF-κB

## Abstract

**Background/Objectives:** Gouty arthritis (GA) is an inflammatory disease caused by increased purine metabolism. The limitations of current anti-GA therapies remain a major challenge. Astilbin, the main flavonoid in Smilax glabra Roxb., was found to exert potential anti-GA effects in our previous study. **Methods:** In this study, a mouse model of monosodium urate (MSU)-induced arthritis and an inflammatory model using mouse bone marrow-derived macrophages (BMDMs) were established. **Results:** Our data showed that astilbin reduced MSU-induced joint swelling and inflammatory infiltration in mice, restored lipopolysaccharide (LPS)/MSU-induced reductions in cell viability, and inhibited the expression levels of inflammatory factors IL-1β, IL-6 and TNF-α. Further studies showed that astilbin significantly reduced MSU-induced increases in NLRP3 and P-p65 protein levels, as well as the expression of ASC, P-IKKα, P-IκBα, and cleaved-caspase-1. **Conclusions:** This study suggests that astilbin may be a promising natural product for the treatment of GA by inhibiting the activation of the NLRP3 inflammasome and NF-κB signaling pathway.

## 1. Introduction

GA is an inflammatory disease caused by the deposition of MSU crystals in the joints, associated with purine metabolism disorders, and characterized by joint swelling, pain, and recurrence [1]. The incidence of gout is increasing every year due to diets high in purines and protein [2]. Promoting uric acid excretion, reducing uric acid synthesis, and exerting anti-inflammatory and analgesic effects are the main directions for the development of drugs to prevent and treat gout, but the complex side effects of existing drugs limit their application [3]. Thus, there is an urgent need to identify safer and more effective therapeutic alternatives.

*Smilax glabra* Roxb. has a long history of use as a raw material for herbal teas and pastries in China, with the effects of clearing heat and eliminating dampness. Dampness-heat is recognized as the TCM pathogenesis of gouty arthritis, which is consistent with the efficacy of *S. glabra.* Meanwhile, our previous studies, along with those conducted by other scholars, have confirmed that *S. glabra* exerts preventive and therapeutic effects on GA, among which astilbin is one of the main active components [4,5,6]. Our previous study isolated six flavonoids from *S. glabra*, including astilbin, neoastilbin, isoastilbin, neoisoastilbin, engeletin and (−)-epicatechin, and compared their antioxidant and anti-inflammatory activities [7]. Among them, the four stereoisomers (astilbin, neoastilbin, isoastilbin, neoisoastilbin) share the same taxifolin-3-O-rhamnoside skeleton but differ in C-2 and C-3 configurations, which affect their anti-inflammatory potency. Although trans-isomers exhibited stronger anti-inflammatory activity, astilbin was the most abundant component (18.10%) and has been recognized as the major bioactive marker of *S. glabra*. Therefore, we selected astilbin for further mechanistic investigation in GA models. Existing studies have suggested that astilbin may serve as the material basis for the anti-gout effect of *S. glabra* and has the potential to be used as a dietary supplement for the prevention and treatment of GA, however, whether it possesses new anti-gout mechanisms remains to be explored.

Immune dysregulation, inflammation, and metabolic disorders are involved in the pathological process of GA [8]. Previous studies have shown that MSU, as a danger signal, can activate the NLRP3 inflammasome and trigger inflammatory responses [9]. In addition, Toll-like receptors (TLRs) recognize endogenous danger signals such as MSU crystals or proteins released from apoptotic cells, leading to activation of the NF-κB signaling pathway, which in turn induces inflammatory mediators and adhesion molecules and upregulates the transcription of inflammatory cytokines (e.g., IL-1β), ultimately resulting in their production and extracellular release [10]. IL-1β also continuously activates the NF-κB cascade through membrane receptors, amplifying the inflammatory response and exacerbating disease progression. Therefore, the NLRP3 inflammasome and NF-κB inflammatory signaling pathway are critical for exploring anti-GA drugs. Thus, the MSU-induced mouse arthritis model and bone marrow-derived macrophages inflammatory model were used in this study to investigate the therapeutic effect and mechanism of astilbin in GA. Notably, while astilbin has been reported to modulate the NLRP3/NF-κB pathway in other diseases, its role in gouty arthritis has not been established. Our study provides the first evidence that astilbin alleviates MSU-induced gouty inflammation through this pathway, supported by inhibitor-based causal validation and a direct comparison with colchicine.

## 2. Materials and Methods

### 2.1. Chemicals and Reagents

Macrophage colony-stimulating factor (M-CSF), trypan blue, monosodium urate, DMSO were purchased from Sigma-Aldrich (St. Louis, MO, USA). Astilbin was purchased from Sichuan Weikeqi Biological Technology (Chengdu, China), with the purity of more than 98%. Colchicine was purchased from Aladdin Reagent Co., Ltd. (Shanghai, China). β-Mercaptoethanol, Cell Counting Kit-8 were provided by Amresco (Washington, DC, USA). Lipopolysaccharide (LPS) was purchased from Solarbio Life Sciences (Beijing, China), Dulbecco’s modified Eagle’s medium (DMEM), fetal bovine serum (FBS) was purchased from Gibco (Carlsbad, CA, USA). Interleukin-1β (IL-1β) enzyme-linked immunosorbent assay (ELISA) kit, IL-6 ELISA kit, and TNF-α ELISA kit were purchased from Mlbio (Boston, MA, USA). CD11b-BB515, F4/80-PE were purchased from BD (Franklin Lakes, NJ, USA). IKKα (1:1000), caspase-1 (1:1000), NLRP3 (1:1000), Phospho-NF-κB p65 (1:1000), IκBα (1:1000), Phospho-IκBα (1:1000), ASC (1:1000), Phospho-IKKα (1:1000), NF-κB p65 (1:1000), pro-caspase-1 (1:1000), Histone H3 (1:1000), β-actin (1:500) antibodies were purchased from Cell Signaling Technology (Boston, MA, USA). IRDye 680LT Goat anti-Mouse, IRDye 800CW Goat anti-Rabbit were from LI-COR (Lincoln, NE, USA).

### 2.2. Establishment of the Mouse Model of Gouty Arthritis

After one week of adaptive feeding, 50 male C57BL/6 mice (6–8 weeks old) were randomly allocated to five groups using a computer-generated random number sequence (Microsoft Excel): normal control, model, colchicine (1 mg/kg), astilbin low-dose (25 mg/kg), and astilbin high-dose (50 mg/kg) groups, with 10 mice per group (Animal Production Certificate No.: SCXK (Shanghai, China) 2017-0005; Animal Use Permit No.: SYXK (Zhejiang, China) 2018-0012). In our study, the sample size of *n* = 10 per group for in vivo experiments was determined based on established practices in the field and the scientific literature, rather than a formal a priori power calculation. All mice were housed in a specific pathogen-free (SPF) laboratory animal facility under controlled conditions: constant temperature and humidity, and a 12-h light/dark cycle. Adequate food and water were provided ad libitum throughout the experiment.

Mice were orally administered astilbin (25 or 50 mg/kg), colchicine (1 mg/kg), or vehicle (normal saline) once daily for 7 consecutive days. On day 6, one hour after gavage, an MSU suspension (100 mg/kg) was injected into the ankle joint of each mouse (except those in the normal control group) at a 45° angle along the dorsal side of the right ankle to establish an acute gouty arthritis model. Analgesia was not used post-injection to avoid interference with the inflammatory response assessment. The normal control group received an equal volume of normal saline in the same manner. Joint swelling was measured at baseline (0 h, immediately before injection) and at 2, 4, 6, 10, and 24 h after MSU injection. At 24 h post-injection, mice were euthanized, and ankle joint tissues were collected for histopathological and Western blotting analyses. The exact sample sizes (*n*) for each experimental group are now explicitly stated in the figure legends.

All animal experiments were approved by the Institutional Animal Care and Use Committee (IACUC) of Zhejiang Chinese Medical University and conducted in accordance with the institution’s guidelines and protocols. No specific inclusion or exclusion criteria were established a priori for animals or experimental units. All animals that successfully underwent MSU injection were included in the analysis. Animals that exhibited signs of severe distress or injection failure (e.g., MSU suspension leakage from the injection site) were excluded. No animals met these exclusion criteria during the experiment.

### 2.3. Cell Culture and Treatment

Bone marrow-derived macrophages (BMDMs) from male mice were isolated and resuspended in complete Dulbecco’s Modified Eagle Medium (DMEM), supplemented with 10% fetal bovine serum (FBS) and 1% penicillin-streptomycin. Erythrocytes were lysed using red blood cell (RBC) lysis buffer, and the cell suspension was filtered through a 40-μm cell strainer to remove cell debris. After centrifugation of the filtered cell suspension, the collected cells were seeded into culture dishes with complete DMEM containing 20 ng/mL macrophage colony-stimulating factor (M-CSF), and cultured in a cell incubator.

The experiment was divided into seven groups: control group (no drug treatment), model group (LPS 500 ng/mL treatment for 6 h, MSU suspension 100 μg/mL treatment for 4 h), positive drug group (pre-protection with colchicine for 0.5 h before MSU addition), low-dose astilbin group (pre-protection with 5 μM astilbin for 0.5 h before MSU addition), medium-dose astilbin group (pre-protection with 10 μM astilbin for 0.5 h before MSU addition), high-dose astilbin group (pre-protection with 20 μM astilbin for 0.5 h before MSU addition). Each group was subjected to three parallel experiments. For the in vitro experiments, BMDMs were pretreated with astilbin (5, 10, or 20 μM) or colchicine (0.1 μM) for 30 min, followed by stimulation with LPS (500 ng/mL) for 6 h and then MSU (100 μg/mL) for an additional 4 h. Cells and culture supernatants were collected immediately after MSU treatment for subsequent analyses.

### 2.4. Cell Viability Assay

Cell viability was assessed using the Cell Counting Kit-8 (CCK-8) assay. Briefly, BMDMs were digested with trypsin and centrifuged at 1000 rpm for 5 min. The collected cells (not supernatant) were resuspended in complete DMEM supplemented with 10% fetal bovine serum (FBS) to a final density of 5 × 10^4^ cells/mL, and 100 μL of this cell suspension was seeded into each well of a 96-well plate. Prior to the CCK-8 reaction, the culture medium in each well was aspirated, and 100 μL of DMEM (containing 10% CCK-8 solution) was added to each well; the plate was then incubated for 1 h. Subsequently, the absorbance at 450 nm was measured using a microplate reader. The formula for calculating cell viability was as follows:

$$Cell\;viability(\%)=\frac{{\mathrm{OD}}_{\mathrm{sample}}-{\mathrm{OD}}_{\mathrm{blank}}}{{\mathrm{OD}}_{\mathrm{control}}-{\mathrm{OD}}_{\mathrm{blank}}}\times 100\%$$

### 2.5. Flow Cytometry Analysis

The specific operational procedures were performed as described previously [11]. Briefly, a single-cell suspension was prepared by collecting 1 × 10^6^ BMDMs into a centrifuge tube and filtering the suspension through a 300-mesh sieve. For the experimental groups, the cells were incubated with PE-labeled anti-F4/80 antibody and BB515-labeled anti-CD11b antibody at 4 °C for 10 min. After incubation, the cells were washed twice with phosphate-buffered saline (PBS), resuspended, and transferred to a flow cytometry tube. Finally, the samples were detected using a flow cytometer (CytoFLEX, Beckman Coulter, Mumbai, Maharashtra).

### 2.6. Measurement of Mouse Ankle Joint Swelling

To standardize the criteria for measuring toe volume, a horizontal line was marked 0.5 cm above the mice’s ankle joints using a fading marker prior to MSU injection. The toe volume of each mouse was measured with a toe volume meter at baseline (0 h, immediately before MSU injection) and at 2, 4, 6, 10, and 24 h after model induction. The swelling index was calculated using the following formula:

$$Swelling\;index\;(\%)=\frac{V_{\mathrm{after}\;\mathrm{injection}}-V_{\mathrm{before}\;\mathrm{injection}}}{V_{\mathrm{before}\;\mathrm{injection}}}\times 100\%$$

### 2.7. Histopathological Assessment

The harvested ankle tissue samples were fixed by immersion in 4% paraformaldehyde, decalcified in ethylenediaminetetraacetic acid (EDTA) solution, and embedded in paraffin. After the paraffin blocks cooled and solidified, tissue sections were cut, followed by hematoxylin-eosin (HE) staining. The stained sections were then observed under a light microscope.

For quantitative analysis of inflammatory infiltration, five randomly selected fields per section were captured at ×100 magnification. Images were imported into ImageJ software (version 1.54p) (National Institutes of Health, Bethesda, MD, USA) for processing. Briefly, the blue channel was extracted to isolate hematoxylin-stained nuclei, followed by morphological filtering to remove background signals from dispersed normal cells. Nuclei segmentation was then performed to identify individual nuclei, and the target regions representing inflammatory cell infiltration were selected. The total area of inflammatory infiltration was measured. All quantitative analyses were performed by two independent observers who were blinded to the experimental groups.

### 2.8. Detection of Inflammatory Factors

The ELISA assay was performed following the procedures described previously [12]. Cell supernatants were collected and centrifuged at 3000 rpm at 4 °C for 10 min. For ankle joint tissues, samples were ground in liquid nitrogen to form a powder, which was then mixed with normal saline at a ratio of 1:10 (g/mL). After homogenization using a homogenizer (Roche, Mannheim, Germany) and centrifugation at 3000 rpm at 4 °C for 10 min, the supernatants were collected. The concentrations of three inflammatory cytokines (IL-1β, IL-6, and TNF-α) in the supernatants were measured according to the ELISA kit instructions.

### 2.9. Western Blotting Analysis

Western blotting was performed to quantify the protein expression levels of P-p65, P-IκBα, P-IKKα, p65, IκBα, IKKα, NLRP3, ASC, pro-caspase-1, and cleaved-caspase-1.

Total protein was extracted from samples using RIPA lysis buffer (supplemented with protease and phosphatase inhibitors), and protein concentrations were determined via the BCA protein assay kit. After adding loading buffer, the protein samples were mixed and boiled for 5–10 min. The proteins were then separated by sodium dodecyl sulfate-polyacrylamide gel electrophoresis (SDS-PAGE) and transferred onto a polyvinylidene difluoride (PVDF) membrane. The membranes were subsequently blocked with 5% skimmed milk in Tris-buffered saline with Tween-20 (TBST) for 2 h at room temperature. Next, the membranes were incubated with the corresponding primary antibodies at 4 °C overnight. After washing with TBST, the membranes were incubated with secondary antibodies. Finally, the protein bands were visualized and scanned using an imaging system. Throughout the experiment, TBST was used to rinse the membranes three times (5 min per wash) after each incubation step.

Western blot band densities were quantified using ImageJ software (National Institutes of Health, Bethesda, MD, USA). The intensity of each target protein band was measured and normalized to the corresponding β-actin band from the same membrane. For phosphorylation proteins (P-IKKα, P-p65, P-IκBα), the phospho-protein levels were normalized to their respective total protein levels (IKKα, p65, IκBα). All quantitative analyses were performed by two independent observers who were blinded to the experimental groups, and the mean values were used for statistical analysis.

### 2.10. Statistical Analysis

All joint swelling measurements and sample collections were performed in a fixed order across groups to minimise procedural variation. All histological and Western blotting analyses were conducted by two independent observers who were blinded to the experimental groups. Statistical analyses were performed using GraphPad Prism 8 software. All data are presented as the mean ± standard deviation (SD). For statistical comparisons, Student’s t-test was used for two-group comparisons, while one-way analysis of variance (ANOVA) followed by either Bonferroni’s or Tukey’s post-hoc test was applied for multiple-group comparisons. A value of *p* < 0.05 was considered statistically significant, and *p* < 0.01 was considered extremely significant.

## 3. Results

### 3.1. Astilbin Exhibits Low Cytotoxicity and Preserves BMDM Viability in MSU/LPS-Induced Inflammatory Models

**Figure 1:**
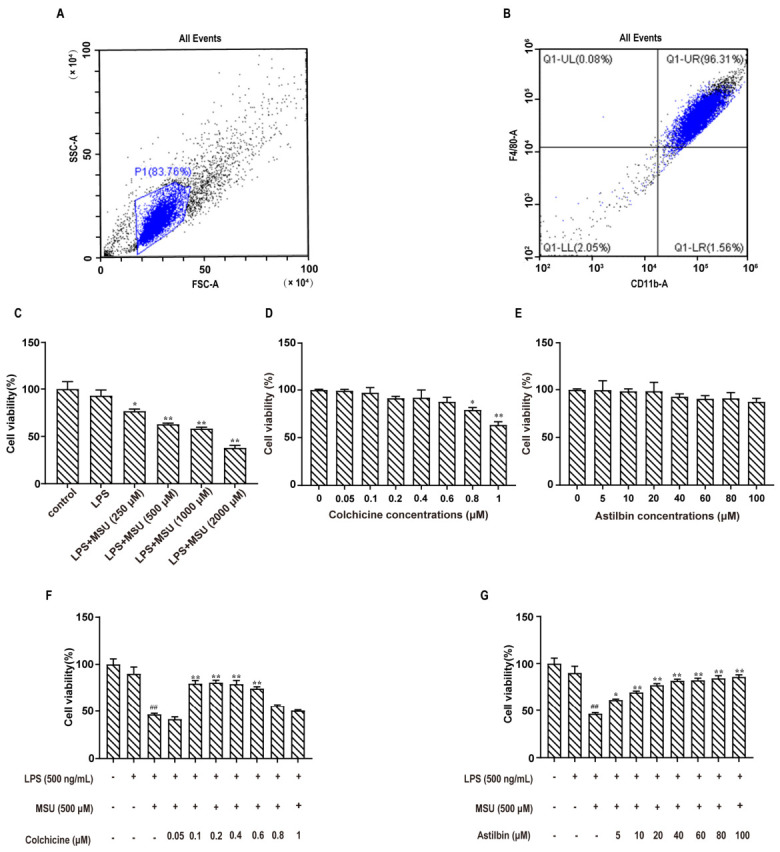
Astilbin inhibits the decrease in cell viability of BMDMs. (**A**,**B**) The purity of the isolated cells was determined by flow cytometry. (**C**) Effect of MSU on the viability of BMDMs. (**D**) Effect of colchicine on the viability of BMDMs. (**E**) Effect of astilbin on the viability of BMDMs. Colchicine (**F**) and astilbin (**G**) effectively alleviated MSU-induced cytotoxicity in BMDMs. Cell viability was determined using the CCK-8 assay kit. Data are presented as mean ± SD of three independent biological replicates (*n* = 3). ^##^ *p* < 0.01 compared with the control group; * *p* < 0.05 or ** *p* < 0.01 compared with the LPS + MSU group.

After M-CSF induction, the rate of F4/80 and CD11b double-positive cells was more than 95% (Figure 1A,B), indicating that the cells obtained met the experimental requirements. To avoid cytotoxicity, the CCK-8 method was used to determine the survival rate of BMDMs after the intervention of different drug concentrations. The results showed that when the concentration of MSU solution reached 250 μM (Figure 1C) or the concentration of colchicine reached 0.8 μM (Figure 1D), the cell survival rate began to decrease, while the astilbin group in the range of 5–100 μM had no obvious decreasing trend, which proved that this range was the safe concentration of astilbin for cells (Figure 1E).

LPS/MSU (L + M) was used to stimulate BMDMs and establish an inflammatory model, simulating the pathogenesis of gouty arthritis in vitro. Inflammatory stimulation led to a decrease in cell viability; however, colchicine (0.1–0.6 μM) and astilbin (5–100 μM) were observed to effectively alleviate MSU-induced cytotoxicity in BMDMs (Figure 1F,G). Notably, cell viability was not further significantly improved when the astilbin concentration exceeded 20 μM. Therefore, for subsequent experiments, astilbin doses were set as 5, 10, and 20 μM (low, medium, and high, respectively), with colchicine used at 0.1 μM.

### 3.2. Astilbin Alleviates Ankle Joint Swelling and Attenuates Inflammatory Infiltration in a Mouse Model of Gouty Arthritis

**Figure 2:**
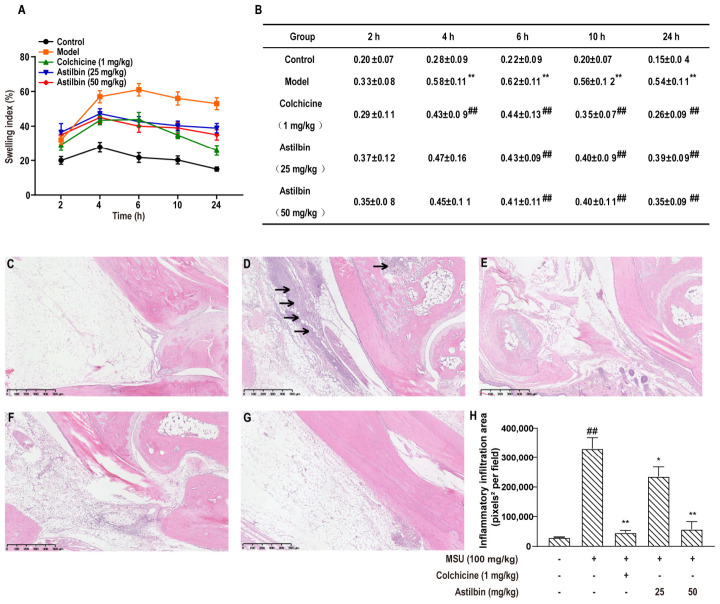
Astilbin Alleviates Ankle Joint Swelling and Reduces Inflammatory Infiltration in Mice with Gouty Arthritis. The swelling degree at different time points is expressed as the ratio of ankle circumference after injection to that before injection. (**A**) Dynamic trend of ankle swelling. (**B**) Quantification of ankle swelling in C57BL/6 mice (%). (**C**–**G**) Effects of astilbin on histologic changes of the ankle joint at 24 h after MSU injection (×100, scale bar: 500 μm). Black arrows indicate inflammatory cell infiltration. (**H**) Quantification of inflammatory infiltration area (pixels^2^ per field). Data represented as mean ± SD of ten mice per group (*n* = 10). * *p* < 0.05 compared with model group; ** *p* < 0.01 compared with model group; ^##^ *p* < 0.01 compared with control group. Note: The swelling index at 0 h is defined as 0% for all groups, as it represents the baseline measurement taken immediately before MSU injection (V_after injection_ = V_before injection_). Therefore, no separate data point is plotted at 0 h.

To investigate the potential anti-inflammatory effect of astilbin, we used a mouse model in which monosodium urate (MSU) crystals were injected into the ankle joints to mimic the acute onset of gouty inflammation. Successful modeling was confirmed by a marked increase in ankle swelling 2 h after MSU crystal injection, and the swelling rate peaked at 6 h in the model group (Figure 2A,B). From 6 h after modeling, significant differences in ankle swelling were observed between the low-dose astilbin group and the model group, as well as between the high-dose astilbin group and the model group (*p* < 0.05). Astilbin treatment not only significantly reduced the degree of ankle swelling but also advanced the peak time of swelling to 4 h after modeling (earlier than that of the model group). These results suggest that astilbin significantly alleviates joint swelling and shortens its duration during the onset of gouty arthritis.

The recruitment of inflammatory cells is a pathological feature of gouty arthritis, so H&E staining was used to observe histological changes and determine inflammatory cell infiltration. In the model group (Figure 2D), synovial epithelial cells exhibited multilayered proliferation, accompanied by massive inflammatory cell infiltration (stained in blue), fibroblast proliferation, and angiogenesis. By contrast, the astilbin treatment groups (Figure 2E–G) showed markedly reduced synovial hyperplasia and inflammatory cell infiltration, with joint tissue architecture better preserved than in the model group. Among these, the high-dose astilbin group (Figure 2G) displayed the most pronounced improvement, with synovial tissue appearing nearly normal and only scattered inflammatory cells remaining. To further quantify the histopathological changes, we measured the inflammatory infiltration area using ImageJ software. As shown in Figure 2H, MSU injection significantly increased the inflammatory infiltration area compared with the control group (*p* < 0.01). Treatment with astilbin at both low (25 mg/kg) and high (50 mg/kg) doses significantly reduced this area in a dose-dependent manner (*p* < 0.05 and *p* < 0.01, respectively). Colchicine (1 mg/kg) also significantly decreased the inflammatory infiltration area, which was consistent with its anti-inflammatory effect.

### 3.3. Astilbin Inhibits Inflammatory Factor Secretion in BMDMs (In Vitro) and Ankle Joints (In Vivo)

LPS stimulation and MSU activation induced the secretion of inflammatory factors in BMDMs, and these factors (IL-1β, IL-6, and TNF-α) can trigger inflammatory cascades, leading to severe inflammatory responses. Thus, we further examined the expression and secretion of IL-1β, IL-6, and TNF-α in two experimental settings: in vitro BMDMs and in vivo ankle joint tissues.

**Figure 3:**
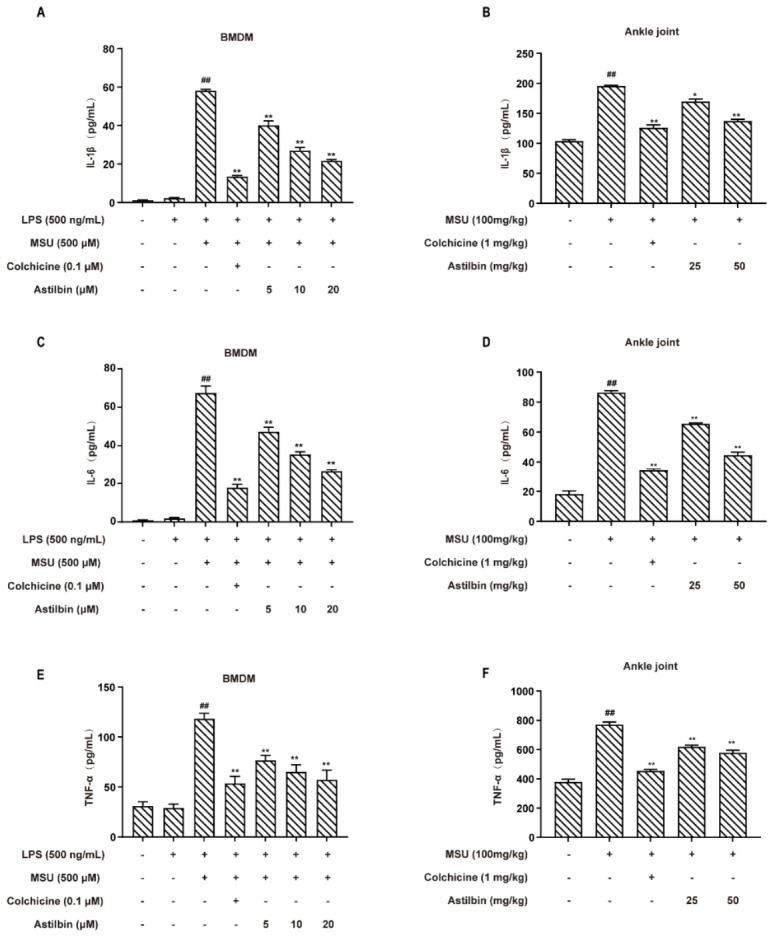
Astilbin inhibits the secretion of pro-inflammatory factors. (**A**,**C**,**E**) Levels of IL-1β (**A**), IL-6 (**C**), and TNF-α (**E**) in the supernatants of BMDMs (treated with LPS/MSU, as described in Methods). (**B**,**D**,**F**) Effects of astilbin on the expression levels of IL-1β (**B**), IL-6 (**D**), and TNF-α (**F**) in ankle joint tissues of MSU-induced acute gouty arthritis mice. Data are presented as mean ± SD of three independent biological replicates (*n* = 3). Each biological sample was measured in triplicate technical replicates. ^##^ *p* < 0.01 compared with the control group; * *p* < 0.05 or ** *p* < 0.01 compared with the model group.

Based on the ELISA results, the secretion of IL-1β, IL-6, and TNF-α was significantly elevated in the in vitro model group (BMDMs treated with LPS/MSU; Figure 3A,C,E). After astilbin treatment, the expression and secretion of these pro-inflammatory factors in BMDMs decreased in a dose-dependent manner. Consistent with the in vitro findings, MSU injection into the ankle joint significantly induced the secretion of IL-1β, IL-6, and TNF-α in in vivo experiments (model group; Figure 3B,D,F). Similarly, astilbin notably reduced the levels of these pro-inflammatory factors in the ankle joint in a dose-dependent manner. These results confirm that astilbin can suppress MSU/LPS-induced pro-inflammatory activation of BMDMs in vitro and alleviate MSU-induced inflammatory responses in the ankle joint in vivo, thereby improving the pathological state of acute gouty arthritis.

### 3.4. Astilbin Inhibits the Activation of NF-κB Signaling Pathway and NLRP3 Inflammasome in BMDMs

MSU crystals may induce the development of gout by stimulating the activation of the NLRP3 inflammasome and the NF-κB signaling pathway. To further confirm whether astilbin exerts its anti-inflammatory effects by blocking the NLRP3 inflammasome and inhibiting the NF-κB signaling pathway, we examined the protein expression of key molecules in both pathways.

**Figure 4:**
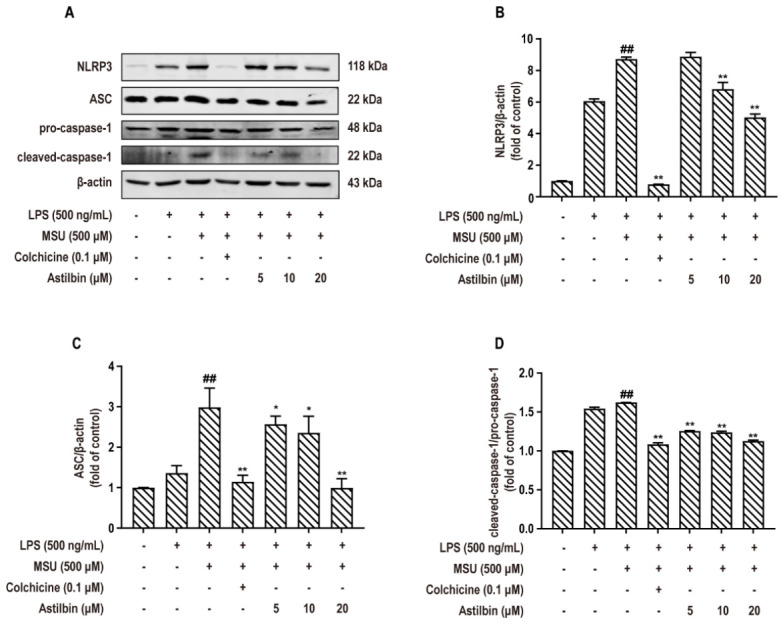
Effects of astilbin on protein expression related to the NLRP3 inflammasome in BMDMs. (**A**) Representative Western blot bands for NLRP3, cleaved-caspase-1, pro-caspase-1 and ASC. (**B**–**D**) Quantitative analysis of the relative protein expression levels of NLRP3, cleaved-caspase-1, pro-caspase-1 and ASC. Data are presented as mean ± SD of three independent biological replicates (*n* = 3). ^##^ *p* < 0.01 compared with the control group; * *p* < 0.05 or ** *p* < 0.01 compared with the model group.

As shown in Figure 4A, LPS priming combined with MSU activation led to a notable increase in the protein levels of NLRP3, ASC, and the cleaved form of caspase-1 in BMDMs. Quantitative analysis (Figure 4B) revealed that the relative expression of NLRP3 normalized to β-actin was significantly elevated in the model group (LPS + MSU) compared to the control group, and astilbin treatment at concentrations of 5, 10, and 20 μM dose-dependently decreased this elevated NLRP3 expression. Similarly, the ASC/β-actin ratio (Figure 4C) was markedly increased in the model group, and astilbin, especially at 20 μM, effectively suppressed this increase. For the cleaved-caspase-1/pro-caspase-1 ratio (Figure 4D), which reflects the activation level of caspase-1, the model group showed a significant rise, and astilbin treatment in a dose-dependent manner reduced this ratio, indicating that astilbin inhibited NLRP3 inflammasome activation. Additionally, colchicine, used as a positive control, also exhibited inhibitory effects on the expression of these key molecules in the NLRP3 inflammasome pathway.

**Figure 5:**
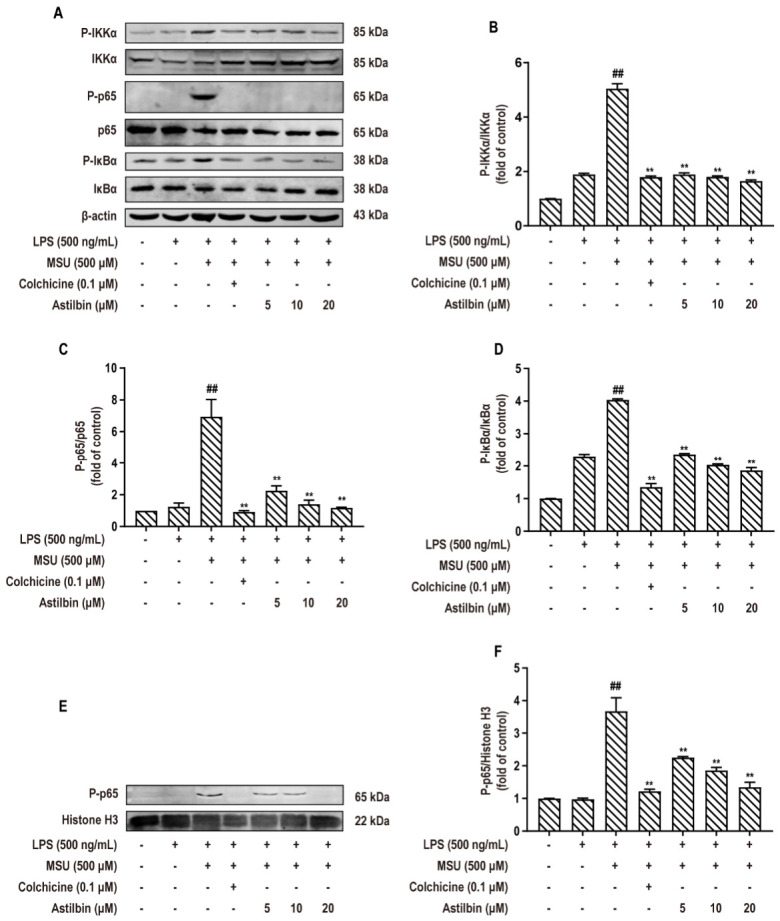
Protein expression related to the NF-κB signaling pathway and NF-κB nuclear translocation. (**A**) Western blot bands for phosphorylated and total IKKα, p65, and IκBα. (**B**–**D**) Quantitative analysis of the phosphorylation ratios (P-IKKα/IKKα, P-p65/p65, P-IκBα/IκBα). (**E**) Protein expression of P-p65 in the nucleus. (**F**) Quantitative analysis of the P-p65/Histone H3 ratio in the nucleus. Data are presented as mean ± SD of three independent biological replicates (*n* = 3). ^##^ *p* < 0.01 compared with the control group; ** *p* < 0.01 compared with the model group.

For the NF-κB signaling pathway (Figure 5), LPS priming combined with MSU activation significantly increased the phosphorylation levels of IKKα (P-IKKα/IKKα, Figure 5B), p65 (P-p65/p65, Figure 5C), and IκBα (P-IκBα/IκBα, Figure 5D) in BMDMs. Moreover, the nuclear translocation of p65, as indicated by the P-p65/Histone H3 ratio (Figure 5F), was also notably enhanced in the model group. In contrast, astilbin treatment at 5, 10, and 20 μM dose-dependently inhibited the increased phosphorylation of IKKα, p65, and IκBα, as well as the nuclear translocation of p65. Colchicine also showed similar inhibitory effects on the NF-κB signaling pathway, consistent with its role as a positive control. These results collectively suggest that astilbin exerts anti-inflammatory effects by blocking the NLRP3 inflammasome and inhibiting the activation of the NF-κB signaling pathway.

### 3.5. Astilbin Inhibits the Activation of NF-κB Signaling Pathway and NLRP3 Inflammasome In Vivo

To investigate whether the NF-κB signaling pathway and NLRP3 inflammasome were involved in the protective effects of astilbin against MSU-induced inflammation in vivo, Western blotting analysis was conducted on ankle joint tissues.

**Figure 6:**
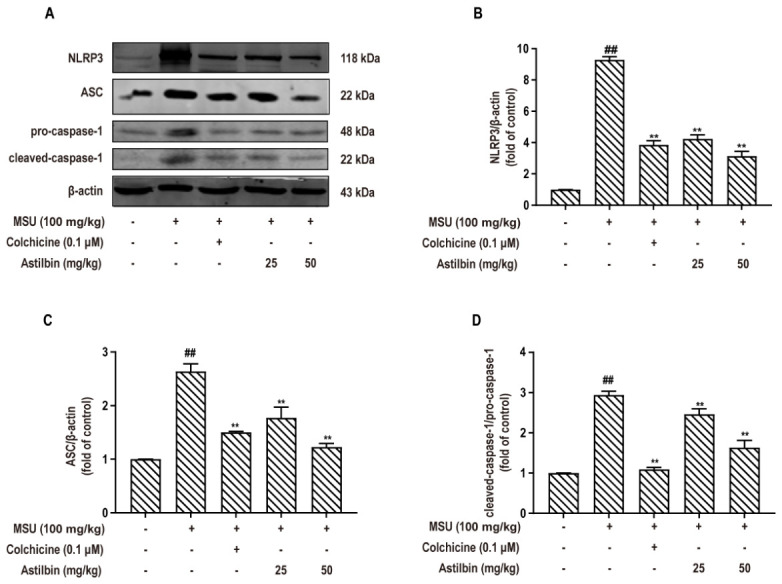
Astilbin inhibits NLRP3 inflammasome activation in vivo. (**A**) Representative Western blot bands for NLRP3, pro-caspase-1, cleaved-caspase-1, and ASC, with β-actin as a loading control. (**B**) Quantitative analysis of the relative protein expression level of NLRP3 normalized to β-actin. (**C**) Quantitative analysis of the relative protein expression level of ASC normalized to β-actin. (**D**) Quantitative analysis of the cleaved-caspase-1/pro-caspase-1 ratio. Data are presented as mean ± SD of three independent biological replicates (*n* = 3). ^##^ *p* < 0.01 compared with the control group; ** *p* < 0.01 compared with the model group.

For the NLRP3 inflammasome, astilbin treatment led to a significant reduction in the protein levels of NLRP3, ASC, cleaved-caspase-1, and pro-caspase-1 in the ankle joints of MSU-challenged mice when compared with the model group (Figure 6A). Quantitatively, the relative protein expression of NLRP3 normalized to β-actin (Figure 6B), the ASC/β-actin ratio (Figure 6C), and the cleaved-caspase-1/pro-caspase-1 ratio (Figure 6D) were all notably decreased in the astilbin-treated groups in a dose-dependent manner.

**Figure 7:**
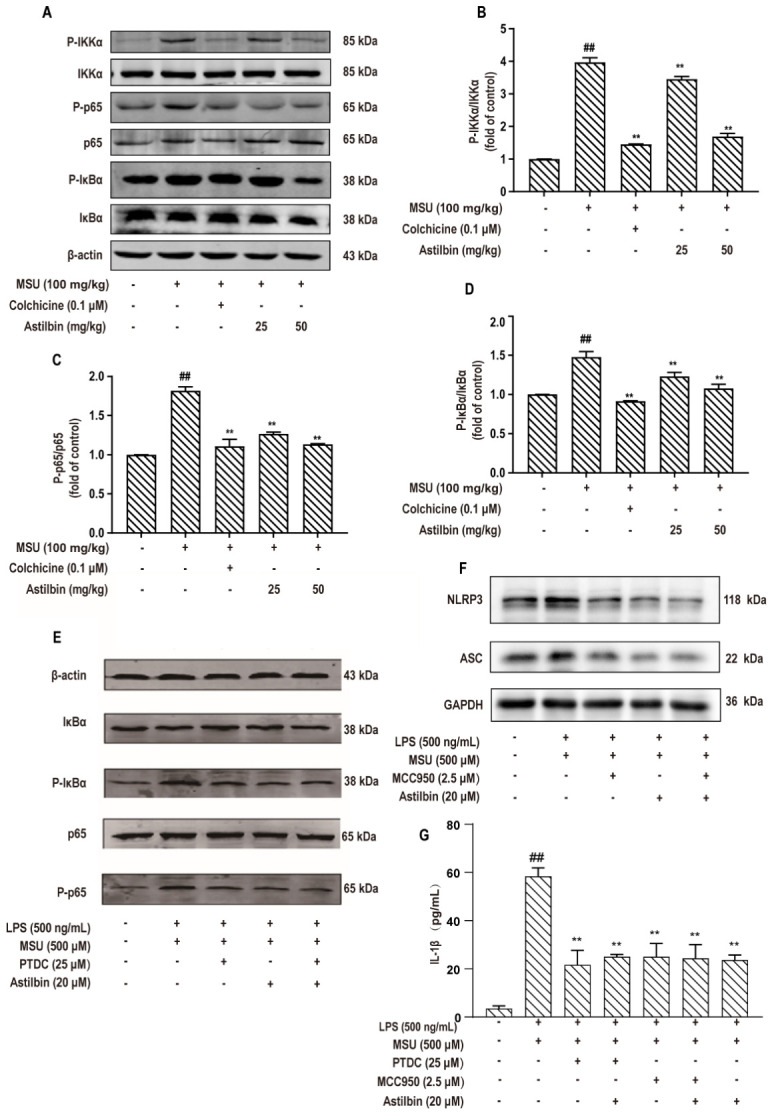
Astilbin inhibits NF-κB signaling pathway activation in vivo and pharmacological inhibition in BMDMs. (**A**) Representative Western blot bands for phosphorylated (P-IKKα, P-p65, P-IκBα) and total (IKKα, p65, IκBα) proteins of the NF-κB signaling pathway, with β-actin as a loading control. (**B**–**D**) Quantitative analysis of the phosphorylation ratios (P-IKKα/IKKα, P-p65/p65, P-IκBα/IκBα). (**E**) Effects of PDTC (25 μM), a specific NF-κB inhibitor, on LPS/MSU-induced phosphorylation of p65 and IκBα in BMDMs. (**F**) Effects of MCC950 (2.5 μM), a specific NLRP3 inhibitor, on LPS/MSU-induced NLRP3 and ASC expression in BMDMs. (**G**) Effects of PDTC and MCC950 on LPS/MSU-induced IL-1β secretion in BMDMs, measured by ELISA. Data are presented as mean ± SD of three independent biological replicates (*n* = 3). ^##^ *p* < 0.01 compared with the control group; ** *p* < 0.01 compared with the model group.

Regarding the NF-κB signaling pathway, the elevated protein expression of P-p65, P-IκBα, and P-IKKα in the model group indicated the activation of the NF-κB signaling pathway. Colchicine, serving as a positive control, and astilbin both effectively inhibited this activation. Furthermore, astilbin significantly lowered the ratios of P-p65/p65 (Figure 7C), P-IκBα/IκBα (Figure 7D), and P-IKKα/IKKα (Figure 7B) compared with the model group, and this inhibitory effect also showed a dose-dependent pattern (Figure 7A–D).

### 3.6. Pharmacological Inhibition of NF-κB and NLRP3 Pathways Confirms Astilbin’s Mechanism of Action

To validate the involvement of NF-κB and NLRP3 inflammasome pathways, we performed pharmacological inhibition experiments using specific inhibitors. As shown in Figure 7E, treatment with PDTC (25 μM), a selective NF-κB inhibitor, significantly reduced LPS/MSU-induced phosphorylation of p65 and IκBα in BMDMs, comparable to the effect of astilbin. Notably, co-treatment with astilbin and PDTC did not result in further suppression, indicating that astilbin and PDTC converge on the same signaling axis. Similarly, MCC950 (2.5 μM), a specific NLRP3 inhibitor, markedly decreased LPS/MSU-induced NLRP3 and ASC expression, mirroring astilbin’s effect, and the combination of astilbin and MCC950 showed no additive inhibition (Figure 7F). ELISA analysis further revealed that both PDTC and MCC950 significantly reduced IL-1β secretion, with no additional suppression when combined with astilbin (Figure 7G). These inhibitor experiments provide causal evidence that astilbin exerts its anti-inflammatory effects through the NF-κB and NLRP3 inflammasome pathways.

## 4. Discussion

The evolutionary loss of uricase leads to elevated uric acid levels in human blood, which promotes the formation and deposition of MSU crystals in joints and adjacent tissues, thereby triggering an intense inflammatory response, namely gouty arthritis [13]. GA is a common form of inflammatory arthritis, characterized by severe joint pain, redness, and swelling [14]. It has emerged as a major public health concern due to its rising incidence—particularly in developed countries—and its association with multiple comorbidities, including cardiovascular disease, chronic kidney disease, and metabolic syndrome [15].

MSU leads to the accumulation of chemotactic macrophages in joints and surrounding tissues, so macrophage activation is closely related to the pathogenesis of GA [16]. This study simulated the occurrence of GA in vitro and in vivo. In the in vitro experiment, we established a method to induce the differentiation of primary monocytes from bone marrow to macrophages by stimulating with 20 ng/mL M-CSF for 7 days and detecting the expression of f4/80 and CD11b by flow cytometry to verify the success of induction. M-CSF plays an important role in the proliferation, differentiation and activity maintenance of monocytes, and can induce monocytes to macrophages [17]. Significant changes in the expression of F4/80 and CD11b as markers of macrophages were observed during macrophage maturation and activation [18]. The results showed that the number of F4/80 and CD11b double-positive cells reached 96.31% after 7 days of M-CSF differentiation, which was consistent with the study results of 95% [19], indicating that this method is feasible to obtain primary mouse macrophages. For in vivo modeling, intra-articular injection of MSU crystals into the ankle joint cavity of mice to mimic the inflammation caused by intra-articular urate deposition during gout flares is a well-established method for screening anti-gout agents [20,21]. We established an acute gouty arthritis model by injecting MSU suspension into the ankle joint of C57BL/6 mice and sham surgery by injecting PBS as a normal control. Compared with the normal group, the toe volume of the model group increased significantly after 2 h (Figure 2A,B) and the inflammatory infiltration of the joint increased (Figure 3), indicating that the modeling method was successful and effective.

Conventional therapeutic approaches for gouty arthritis include nonsteroidal anti-inflammatory drugs (NSAIDs), corticosteroids, colchicine, and urate-lowering therapies (ULTs) such as allopurinol and febuxostat [22]. These approaches primarily focus on symptom management rather than targeting the biochemical pathways underlying hyperuricemia and inflammation. Conventional treatments often have limitations, such as inducing adverse side effects, potential drug–drug interactions, and poor patient compliance [23]. Therefore, the search for safe and effective novel therapeutic agents has become a research focus. Recent studies have demonstrated that dietary polyphenols hold potential for the treatment of gouty arthritis, attributed to their anti-inflammatory, antioxidant [24]. *S. glabra* has a long history of edible use in China. Our previous studies have also shown that flavonoids, a subclass of polyphenols, extracted from *S. glabra* exert anti-inflammatory effects and have been reported to exhibit urate-lowering activities in other models [6,7]. As one of the main flavonoid components in *S. glabra*, astilbin exhibits potential for development as a dietary supplement. It has been reported that astilbin can reduce uric acid in vivo, meanwhile, studies have shown that astilbin can reduce TNF-α, IL-1β and other inflammatory factors in RAW264.7 cells and J774A.1 cells [25,26]. In the present study, we screened the drug concentrations using the CCK-8 assay. The results showed that LPS and MSU significantly inhibited cell proliferation (Figure 1C), whereas astilbin ameliorated LPS- and MSU-induced cell damage within the administered concentration range (Figure 1F,G). Meanwhile, even at a concentration five times higher than the effective dose also exhibited no cytotoxicity (Figure 1E), which confirms the potential of astilbin for development as a dietary supplement. Regrettably, the present study did not involve in vivo toxicity experiments in animals, which will be further conducted in our subsequent research.

MSU crystals can activate the NLRP3 inflammasome in macrophages, leading to the cleavage and release of the proinflammatory cytokine IL-1β [27]. In turn, IL-1β further promotes the release of other cytokines, such as TNF-α and IL-6, as well as the recruitment of neutrophils. This sustains the inflammatory response and contributes to the severe pain and swelling characteristic of gout flares [28]. It is evident that inflammatory cytokines play a crucial role in the occurrence and progression of gout. In the present study, we found that MSU significantly increased the expression levels of IL-1β, IL-6, and TNF-α. This result is consistent with the aforementioned reports, which also confirms the reliability of our experimental model. Further investigation revealed that astilbin significantly reduced the MSU-induced upregulation of IL-1β, IL-6, and TNF-α expression, which is consistent with previous reports [5,25].

The release of IL-1β is primarily regulated by the NLRP3 inflammasome and NF-κB signaling pathway. Accumulation of NLRP3 inflammasomes occurs mainly after MSU is engulfed into the cytoplasm, where ASC and caspase-1 protein interact to cleave the caspase-1 precursor from the complex to generate active caspase-1, which further processes the precursor of IL-1β to produce IL-1β and is released into the extracellular space to cause inflammation [29]. The NF-κB signaling pathway regulates the expression of many inflammatory mediators, adhesion molecules and other proteins, and stimulates the production and release of pro-IL-1β [30]. During activation of the NF-κB pathway, activation of NF-κB dimers is critical for the subsequent series of inflammatory responses. When cells are in a resting state, NF-κB dimers bind to IκBα in the cytoplasm and exist in an inactive state. When cells are stimulated, IκBα begins to be phosphorylated and rapidly degraded by IKKα, releasing activated NF-κB, which then enters the nucleus to bind to DNA and initiate transcriptional target proteins [31]. To investigate whether astilbin regulates IL-1β release and alleviates the occurrence and progression of gouty arthritis through these two signaling pathways, we detected the proteins associated with these pathways. Consistent with the aforementioned studies, compared with the normal group, MSU stimulation increased the expression of proteins related to the NLRP3 inflammasome and NF-κB signaling pathway in both in vitro and in vivo models (Figure 4, Figure 5, Figure 6 and Figure 7). In contrast, astilbin significantly reduced the expression of these proteins in a dose-dependent manner, and similar findings have also been reported in studies on plant extracts containing astilbin [32].

Interestingly, colchicine also inhibited both the NLRP3 inflammasome and the NF-κB pathways in our models, suggesting that its anti-gout mechanism may involve modulation of these pathways beyond its classical microtubule-disrupting activity. Although colchicine has been reported to modulate the NLRP3 inflammasome and NF-κB signaling in other pathological conditions [33], to our knowledge, its role in regulating these pathways specifically in gouty arthritis has not been extensively characterized. Whether colchicine directly targets these pathways in gouty inflammation warrants further investigation. Future studies employing specific pharmacological inhibitors or genetic approaches, such as NLRP3- or p65-knockout models in MSU-induced arthritis, would help clarify whether colchicine’s therapeutic effects in gout are directly mediated through these pathways. However, while colchicine exhibited clear cytotoxicity at concentrations ≥ 0.8 μM, astilbin showed no obvious cytotoxicity even at 100 μM. Moreover, high-dose astilbin (50 mg/kg) demonstrated comparable or slightly superior efficacy to colchicine in reducing joint swelling and inflammatory cytokine production. These findings suggest that astilbin may offer a safer alternative to colchicine by modulating the NLRP3/NF-κB axis without the microtubule-related toxicity. Future studies are warranted to further compare their pharmacokinetic and safety profiles. On the other hand, numerous studies have reported a crosstalk between the NF-κB signaling pathway and the NLRP3 inflammasome [34,35], which was not explored in the present study and will be further investigated in our subsequent research.

To move beyond correlative evidence and establish a causal relationship, we performed pharmacological inhibition experiments using specific pathway inhibitors. PDTC, a well-characterized NF-κB inhibitor, has been shown to suppress phosphorylation of IKKα and NF-κB p65, thereby blocking the canonical NF-κB signaling cascade [36]. In our study, PDTC treatment reproduced the inhibitory effects of astilbin on P-p65 and P-IκBα expression, and the lack of additive effect when combined with astilbin indicates that astilbin acts through the same NF-κB signaling axis. Similarly, MCC950, a potent and specific NLRP3 inhibitor that binds to the NACHT domain and prevents NLRP3 oligomerization, significantly reduced NLRP3 and ASC expression, mirroring astilbin’s effects [37]. The absence of additive inhibition in the combination groups provides strong causal evidence that astilbin’s anti-inflammatory action is mediated through these two pathways. Nevertheless, we acknowledge that pharmacological inhibitors may have off-target effects, and future studies using genetic approaches, such as NLRP3- or p65-knockout models, would further strengthen the causal validation.

Taken together, we confirmed that astilbin has the effect of preventing and treating gouty arthritis, which is related to the NLRP3 and NF-κB pathways. Therefore, astilbin may represent a potential lead compound for the prevention and treatment of gouty arthritis, and exhibits potential for development as a dietary supplement.

## 5. Conclusions

Overall, the present study demonstrates that astilbin, a natural flavonoid compound, significantly inhibited monosodium urate (MSU)-induced gouty arthritis in both in vitro and in vivo models. Its mechanism of action involves inhibiting NF-κB signal transduction and attenuating NLRP3 inflammasome activation, which in turn reduces the secretion of interleukin-1β (IL-1β) and inflammatory infiltration in macrophages, as well as alleviates the swelling of joint synovial tissues. Given its safe source, astilbin may hold potential as a novel dietary supplement for the daily prevention and management of gouty arthritis, although further clinical studies are warranted to validate its efficacy and safety in humans.

## Data Availability

The original contributions presented in this study are included in the article/Supplementary Material. Further inquiries can be directed to the corresponding author.

## Supplementary Materials

The following supporting information can be downloaded at: https://www.mdpi.com/article/10.3390/nu18142360/s1, Figure S1: Original images for Western blots.

## Abbreviations

GA, gouty arthritis; MSU, monosodium urate; BMDM, bone marrow-derived macrophages; ELISA, enzyme-linked immunosorbent assay; CCK8, cell counting kit-8; NLRP3, NOD-like receptor thermal protein domain associated protein 3; NF-κB, nuclear factor kappa-B; TNF-α, tumor necrosis factor-α; IL-6, interleukin-6; IL-1β, interleukin-1β; LPS, lipopolysaccharide; HE, hematoxylin-eosin; ASC, apoptosis-associated speck-like protein containing a card; Caspase-1, cysteinyl aspartate-specific proteinase-1; FBS, Fetal bovine serum; TLRs, toll like receptors.
