# Supplementary material for: Astilbin Alleviates Gouty Arthritis via Regulating NLRP3 Inflammasome and NF-κB Signaling Pathway: A Comprehensive Study on In Vitro and In Vivo Experimental Models
Source: Nutrients. 2026 Jul 18;18(14):2360. doi: 10.3390/nu18142360 (PMC13415446; doi:10.3390/nu18142360)

in vivo

NF-κB

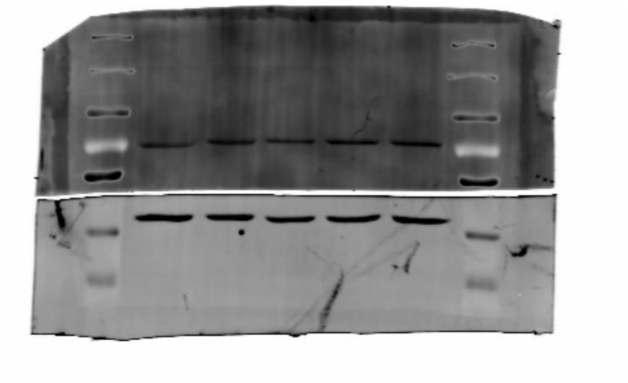

pro-caspase-1 & cleaved-caspase-1

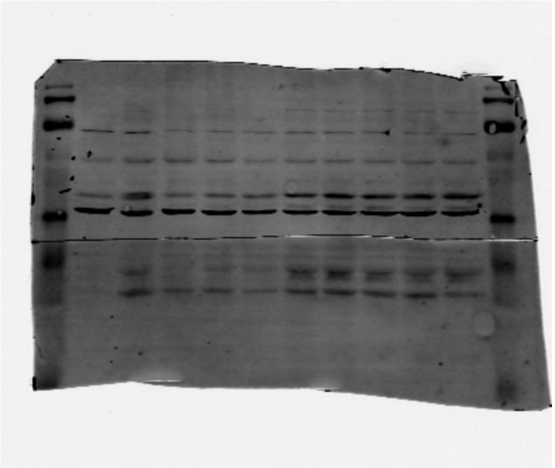

NLRP3

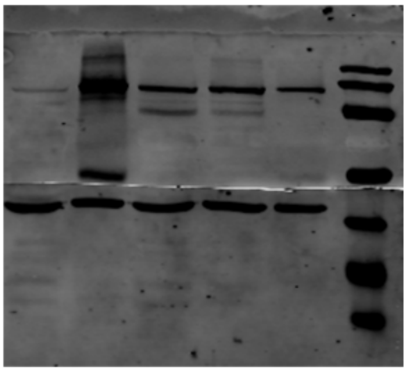

ASC

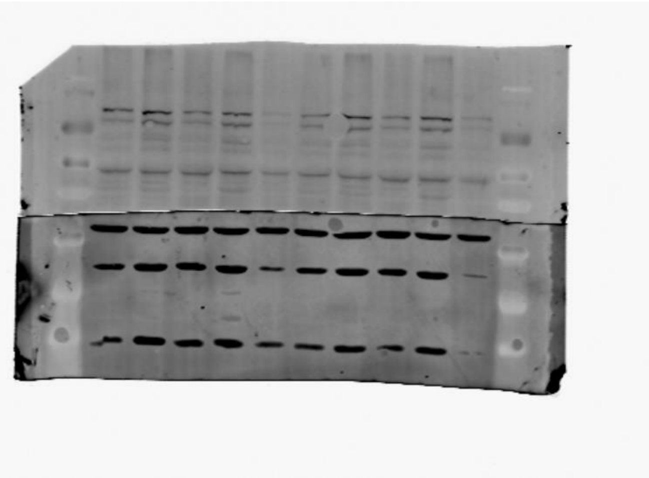

IKBα

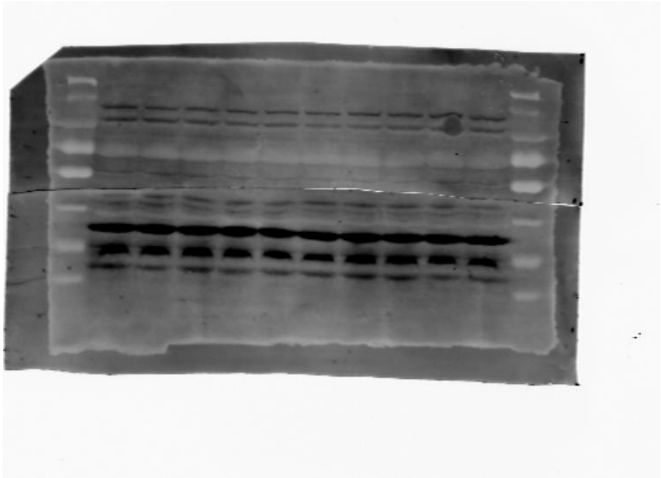

IKK

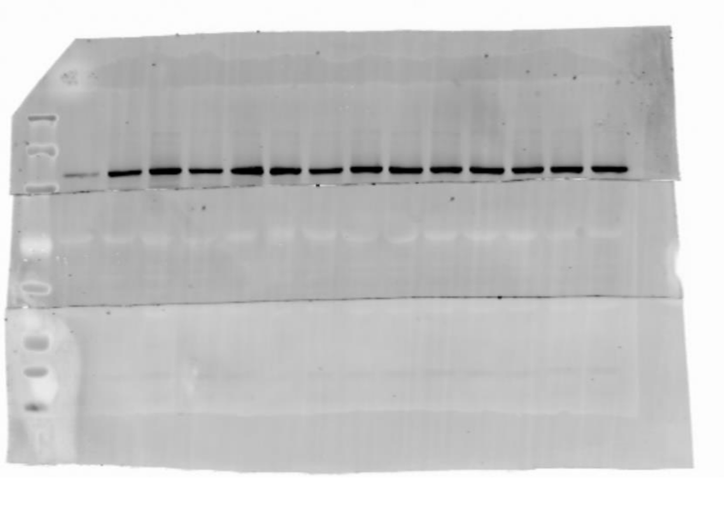

P-IKBα

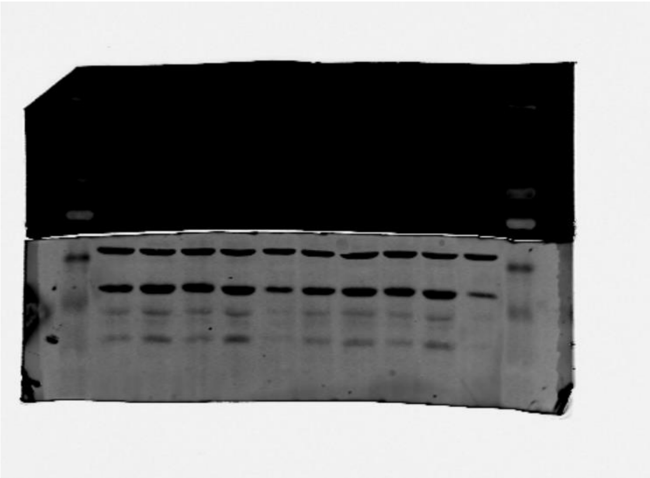

P-IKKα

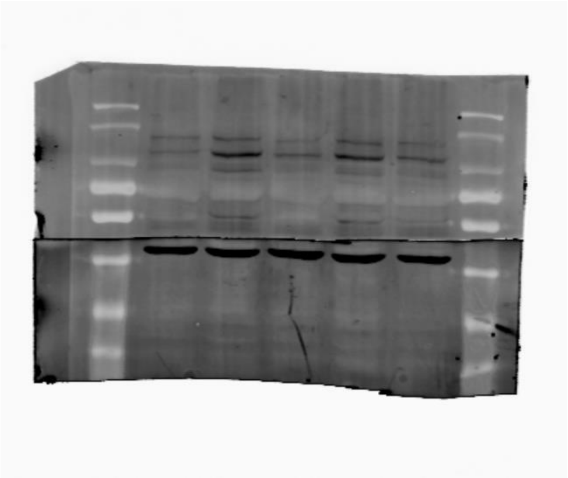

P-p65

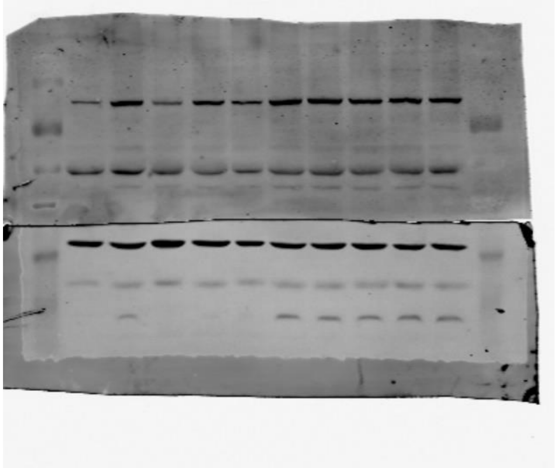

In vitro

NLRP3

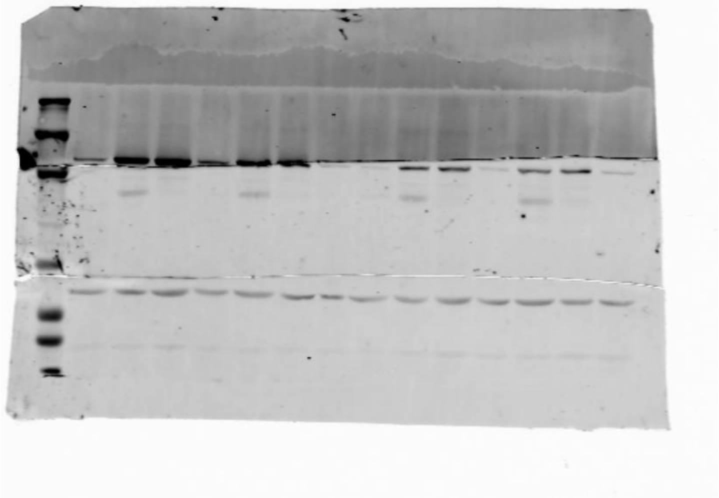

ASC

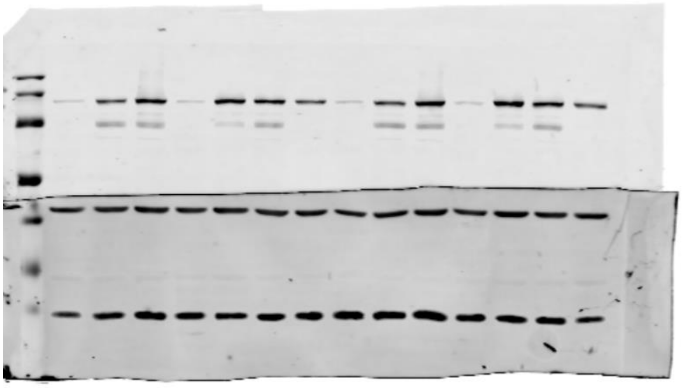

pro-caspase-1 & cleaved-caspase-1

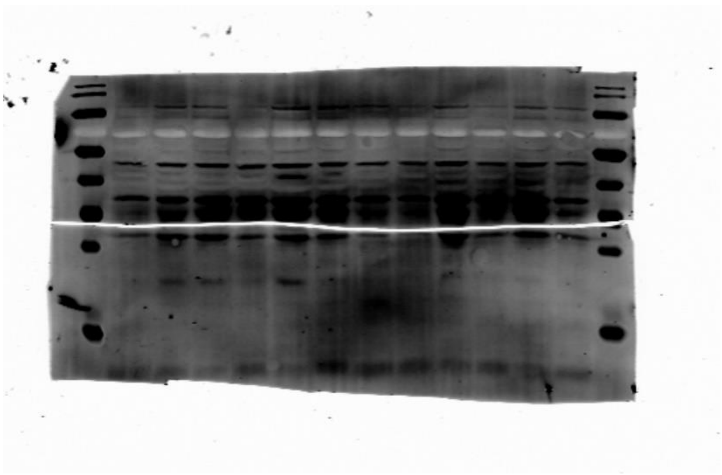

P65

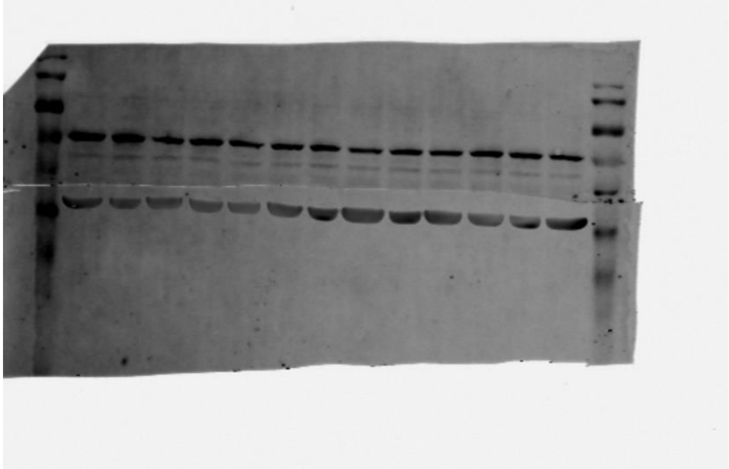

P-P65

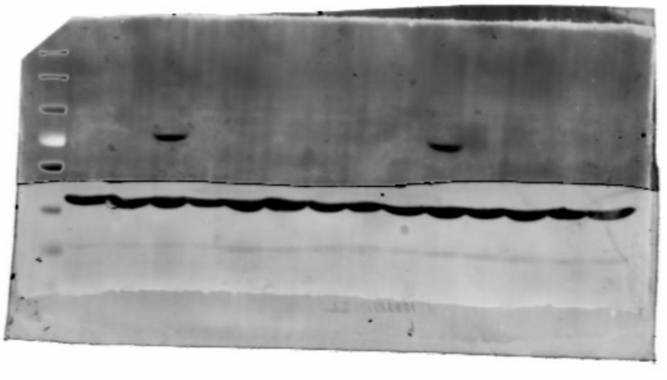

IKK

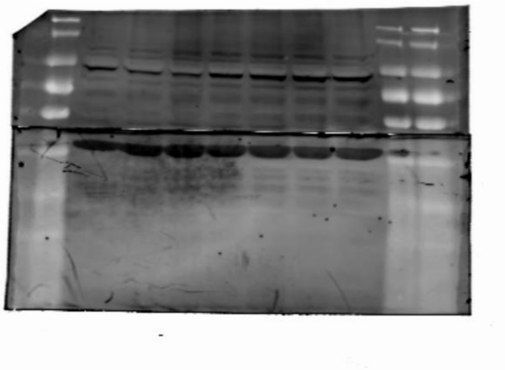

P-IKK

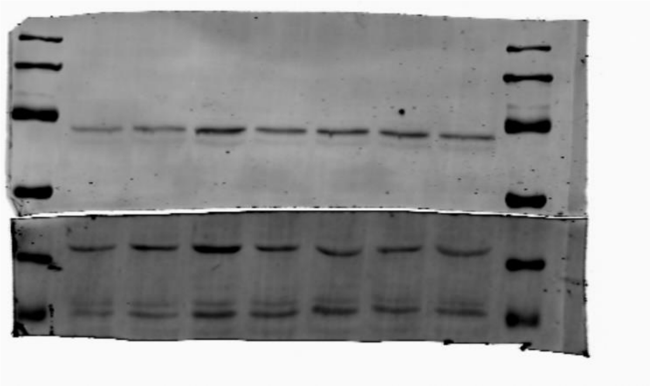

IKB

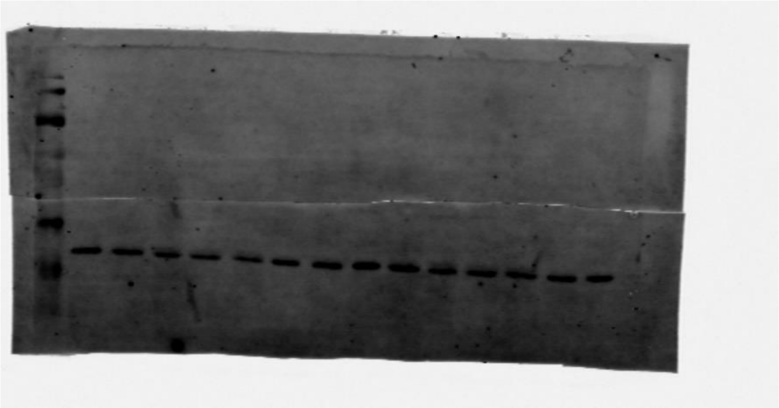

P-IKB

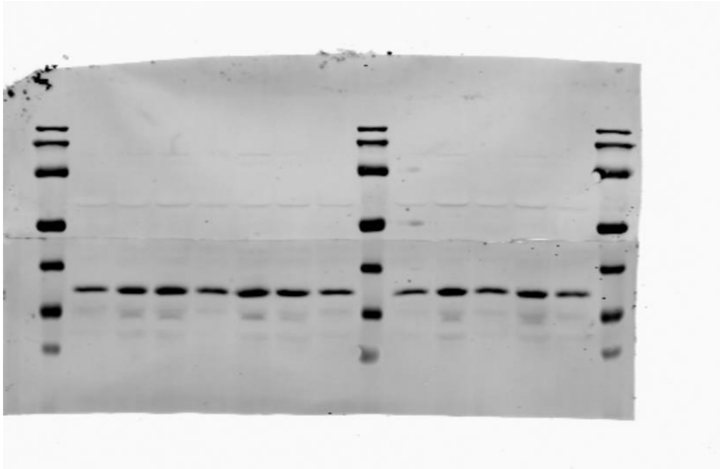

P-P65 & Histon H3

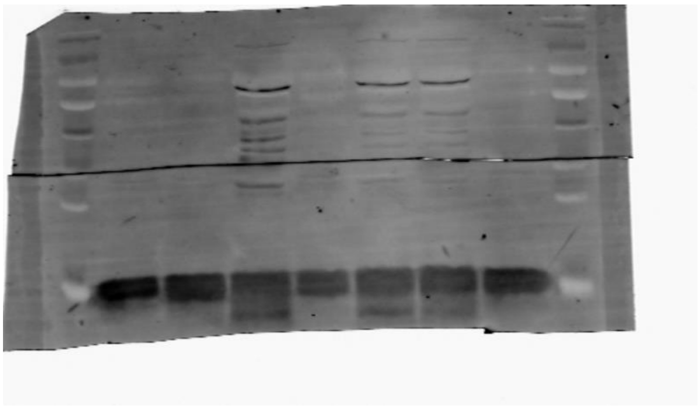

NLRP3、ASC、GAPDH

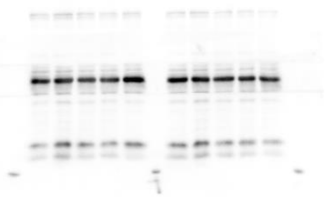

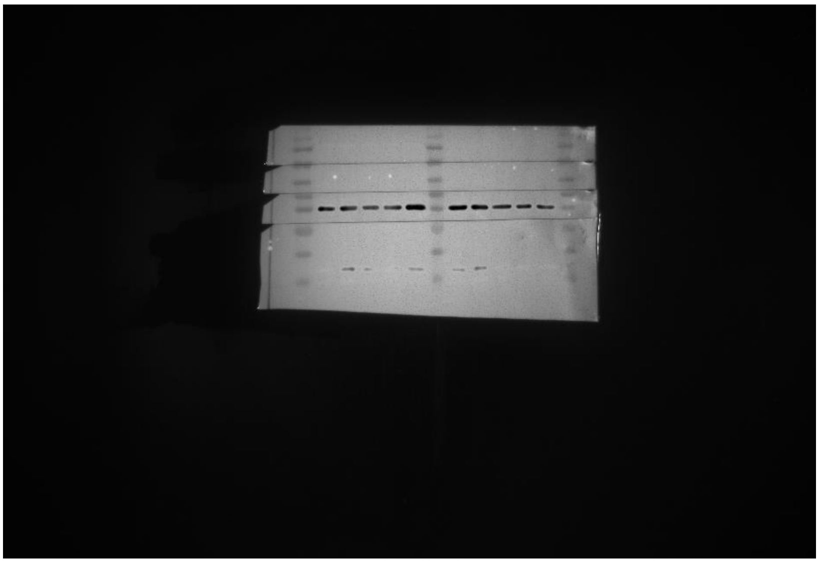

Supplement: Supplementary file 1 [file nutrients-18-02360-s001.zip › nutrients-4383258-supplementary.pdf]
